# Urban environmental exposure as a factor in adaptive neuromodulation: a conceptual framework

**DOI:** 10.3389/fnhum.2026.1939863

**Published:** 2026-08-18

**Authors:** Aurela Qamili, Anja Cenameri

**Affiliations:** Department of Engineering, Albanian University, Tirana, Albania

**Keywords:** artificial intelligence, context-aware neuromodulation, digital exposome, EEG, environmental neuroscience, GIS, local climate zones, neuroarchitecture

## Abstract

Urban neuromodulation and neurofeedback systems have become increasingly adaptive and closed-loop. However, they still focus primarily on brain signals and often ignore the urban areas where these signals happen. The framework introduced in this study integrates EEG, physiological measures, indicators of environmental exposures, and GIS spatial analysis. Our goal is to incorporate Local Climate Zone classification into one unified cycle of user-specific interpretation. Contrary to the typical methodology in digital exposomes and environmental neuroscience, where the individual-to-environment relationship is assessed for exposure or well-being purposes, our approach treats urban exposure directly as an input to neurofeedback adaptation. In this respect, the proposal involves using EEG together with biological signals, along with spatial and environmental exposure metrics. According to the principal hypothesis, the use of spatial and environmental exposure data may help to lower ambiguity in the interpretation of the neurophysiological signal. The article is organized around four interconnected layers. They are environmental exposure, human sensing, AI-based fusion, and adaptive feedback. The layers describe the model through a non-clinical use case and examine major ethical, technical, and social challenges. The suggested architecture is conceptual and non-clinical. The aim is to orient future empirical and translational studies rather than to report already validated outcomes. The main contribution of the framework is the use of urban exposure as an active contextual input for interpreting brain–body states and guiding adaptive feedback, instead of treating the environment as background noise.

## 1 Introduction

Urbanization is changing the context in which the cognitive and emotional conditions are experienced daily. With urbanization, people experience varying levels of exposure to traffic, noise, air pollution, heat, social crowding, a lack of green space, and a visually complex environment. Exposure to all these factors not only involves the urban-planning and environmental context but also alters the regulation of stress, attention, affect, and mental health. Thus, current research in urban mental health has begun to focus on how the brain responds to such exposures, using mobile EEG and other physiological sensing techniques (Senkler et al., 2025; Mavros et al., 2022; Manohar and Kurukkanari, 2026).

This relatively new domain, commonly discussed within neurourbanism and environmental neuroscience, suggests that certain neurophysiological and psychological responses can be observed in metropolitan areas. The use of mobile EEG demonstrated that brain activity can differ depending on whether one is in green areas, crowded places, indoors, outdoors, or urbanized zones. Physiological measures like electrodermal response and changes in heart rate can be used to analyze general state and stress reactions (Mavros et al., 2022; Senkler et al., 2025). Moreover, it is found that green spaces help induce relaxation and stress recovery. However, it may depend on environmental conditions, exposure time, sensory experiences, and the type of green area (Ma et al., 2026). However, actual data on this topic remains quite heterogeneous, and many studies lack a proper description of their urban exposures (Senkler et al., 2025).

Recent empirical work also shows that restorative effects are not uniform across all green or blue environments. Yao et al. (2023) assessed physiological efficiency and benefit thresholds in various urban environments using EEG and heart rate parameters. In addition, it was demonstrated that recovery responses may follow a threshold-related instead of a strictly linear pattern of exposure. Yang et al. (2025) showed that green spaces can become not a source of benefits but a stress factor with the degradation of environmental quality. This proves that the process of restoration cannot only depend on the presence of green areas but also on their ecological and sensory state.

At the same time, neuromodulation and neurofeedback are currently being developed in dynamic and personalized forms. The biomarkers do not rely on the context in which these markers occur. This could be an important limitation when it comes to the application of such systems in real-life scenarios, as stress and mental workload, as well as the process of recovering from them, cannot be caused solely by internal neural mechanisms. They depend on location, environment, and experience in the environment. Information on the environment, physiology, behavior, perception, and geotagging can be integrated into model interactions between individuals and environments (Johnson et al., 2023). The same suggestion has been made in the field of environmental neuroscience, where the integration of ambient exposures, physiological measurements, and brain biomarkers should be used for studying mental flexibility in real-life environments (Morina, 2026). Existing digital exposome approaches provide important tools for measuring person–environment interactions. However, they usually end with exposure assessment, wellbeing estimation, or cognitive-load modeling. The gap is how such contextual information can be translated into adaptive feedback decisions. This is where the present framework differs: it does not only ask how urban exposure affects brain and body states, but how those contextual signals could be used to guide non-invasive, real-time neurofeedback in a safe and personalized way.

In this article, neuromodulation is used as the broader term for approaches that aim to influence neural activity through feedback, stimulation, or adaptive regulation. Neurofeedback is treated as a specific non-invasive form of neuromodulation in which users receive real-time information about brain activity and learn to regulate selected neural states. The proposed framework focuses primarily on non-invasive EEG-based neurofeedback, while neuromodulation is used as the wider conceptual field in which this approach is situated.

Spatial intelligence may be useful in filling this gap. Geographic Information Systems, environmental mapping, and the Local Climate Zone (LCZ) mapping method could quantify an urban context using variables such as building density, surface texture, green space distribution, heat exposure, and environmentally responsive urban morphology. In Tirana, LCZ mapping has been applied to identify environmentally weak points and promote climate-responsive planning (Cenameri and Albert, 2021). As part of a wider trend, as Han et al. (2024) note, of using LCZs to analyze urban climate and guide sustainable city planning more generally. This is where spatial intelligence becomes useful for the framework proposed here: it provides the link between an individual’s physiological and brain-state monitoring and the specific urban conditions they are actually exposed to. Gál et al. (2026) conducted a structured review of LCZ applications. They note that the framework has become central to urban climate simulation. This occurs because it captures the heterogeneity of urban form more effectively than earlier classification systems, making it well suited to multiscale modeling.

The primary gap addressed in this paper is the integration of three research tracks that have remained largely independent: artificial intelligence-based neuromodulation, EEG-based urban mental state monitoring, and GIS-based environmental exposure assessment. Although neuromodulation systems have become more adaptive and urban neuroscience has grown more contextual, these domains have not yet been fully integrated. This paper proposes that future non-clinical neuromodulation and neurofeedback systems should evolve from brain-only to brain–body-environment adaptation.

This paper provides a theoretical AI model for context aware neuromodulation in urban environments. The model utilizes the combination of EEG brain state detection, physiological monitoring, exposure to environment and GIS based spatial intelligence. Its unique contribution is the acknowledgment of the urban exposure context as a key element informing brain state interpretation and subsequent neurofeedback/ neuromodulation. No empirical evidence is provided. We outline a future-oriented design motivated by recent advances in AI-based neuromodulation, environmental neuroscience, mobile EEG, and exposure modeling. The DigitalExposome framework (Johnson et al., 2023) and the Leef model (Morina, 2026) use models to estimate person-environment interactions in terms of exposures or mental well-being, but the current framework is different. It uses environmental exposure as an input in a feedback loop to guide neurofeedback or neuromodulation.

The primary hypothesis is that the use of an exposure-informed model will assist in interpreting neurophysiological states due to increased clarity in the interpretation of brain–body signal. EEG, HRV, and EDA signal may denote states of arousal, attentional engagement, cognitive load, and other recovery related activities. However, there is no intrinsic interpretability associated with these measures. It is possible that one and the same physiological state reflects traffic-induced stress, exercise, mental concern, social engagement, or focused attention. Information related to the spatial and environmental factors does not substitute neurophysiological measures but only constrains possible interpretations for the model.

## 2 Recent advances within AI-driven neuromodulation

Neuromodulation has been developed using predefined stimulation or feedback schemes, in which parameters are chosen in advance and adjusted at set intervals. Although this paradigm of open-loop neuromodulation has found numerous valuable and effective uses in medicine and research, it is quite limited in cases involving dynamic changes in brain states over time, across situations, and in response to personal factors. Recently, the trend toward closed-loop and adaptive approaches has gained attention. Neural or physiological signals are collected and studied in real time for feedback and stimulation. Artificial intelligence is central to this transition because it enables real-time analysis, prediction, and personalization of neural data. It makes possible the analysis, recognition, prediction, and personalization of neural data (Yang et al., 2024).

The transition from open-loop to closed-loop neuromodulation is offered as a move directed to more personalized brain stimulation. Adaptive algorithms and machine learning can modify intervention parameters as needed to change neural or physiological markers (Kachhadia et al., 2025). Du et al. (2025) further this by proposing multimodal brain-state decoding that combines EEG, fMRI, and fNIRS with machine learning. This approach can support personalized and closed-loop interventions. Where static interventions ignore the person’s current state, adaptive methods work from biomarkers or extracted features, using that data to decide when and how to intervene. This types of neuromodulations through AI can avoid unnecessary stimulation and facilitate rapid decision-making (Yang et al., 2024). However, most of these studies have been clinical, but their underlying principles involve neurofeedback that is not necessarily clinical. Adaptive mechanisms need state estimation, proper timing of feedback, and individual response modeling.

Even with advances in neuroimaging techniques, EEG remains one of the most promising non-invasive methods for measuring brain states. Its accessibility, portability, and the possibility of real-time signal processing make it a prominent technology. EEG-based solutions allow for the registration of changes in oscillatory states of the brain associated with attention, relaxation, fatigue, workload, and emotional regulation. Traditionally, EEG has used manually designed features such as power in different frequency bands, including theta, alpha, beta, and gamma. However, with new possibilities in machine and deep learning approaches, it is possible to perform feature extraction more easily.

Recent advances in AI algorithms for EEG analysis include classical machine-learning classifiers, CNNs, RNNs, and transformer models. Transformer models are of particular interest because they can model long dependencies and contextual connections in the sequential and time-frequency analyses of neural data. Visual transformers were used in a study by Afzal et al. (2025) to classify electrographic seizures in intracranial EEG spectrograms, showing that deep learning algorithms can increase scalability in interpreting large quantities of neural data. Such a clinical study serves as an example of a general method: AI translates complex EEG data into interpretable information. The related literature has also examined AI and computer-science methods of EEG signal classification, emphasizing the importance of algorithm choice in EEG states’ interpretation (Qamili and Kola, 2024).

Neurofeedback is another promising approach within non-invasive neuromodulation. Neurofeedback allows a person to receive real-time information about their brain activity and train themselves to change the state of the neural network using that feedback. This way, neurofeedback becomes especially important for non-clinical uses linked with attention, relaxation, cognitive control, stress, and self-regulation. Real-time neurofeedback has been introduced as a non-invasive method for regional neuromodulation of neural activity. Nevertheless, it is influenced by methodological factors, including feedback modality, target signal, training protocol, and the ability of the participant to learn to control the neural activity (Galang et al., 2025). Experiments on the use of EEG-fMRI have been performed. It was found that there is a potential correlation between quick electrophysiological activity and spatially localized brain activity. This, however, requires specific equipment and cannot be widely used in practice (Ciccarelli et al., 2023).

The trend toward wearable and embedded EEGs is very important and an advantage for expanding neuromodulation and neurofeedback beyond the lab setting. The latest reviews of in-ear EEG technology emphasize the trend toward developing small-sized, wireless, and multimodal wearable devices with embedded processing and machine learning capabilities to allow brain state assessment under ambulatory conditions (Channa et al., 2026). Such developments are important since context-aware neuromodulation relies on signals that could be obtained outside of the experimental setting. In addition, wearable EEG makes it possible to merge neural signals with physiological parameters.

While all these advancements have been made, there has not been significant progress in making AI-based neuromodulation more sensitive to its environment and context beyond simply being responsive to neural data and device adaptivity. Indeed, the most common framework in use is still signal collection, brain state classification, and feedback modification based on that. However, while it is an essential step forward, it ignores the fact that brain states develop within particular contexts and under specific conditions. The same pattern picked up by the EEG in a quiet, peaceful environment is unlikely to receive the same interpretation in a loud, crowded urban area.

Such a constraint highlights the need for a framework that integrates urban environmental data, thereby making adaptive neuromodulation more effective in real-world conditions.

## 3 Urbanization as a neurophysiological context

Urbanization leads to situations in which individuals’ cognitive and affective states are permanently affected by their exposure to the environment. The experience of living in an urban area, especially a densely populated one, is characterized by exposure to traffic noise, crowding, visual stimuli, air pollution, thermal stimuli, and a lack of green spaces and recovery. These stimuli are not independent of each other. They combine to create exposure conditions that may affect processes related to stress, attention, arousal, fatigue, and subjective well-being.

Urban exposure should not be treated as a set of isolated variables. In real environments, heat, noise, traffic density, air pollution, crowding, and visual complexity often occur together, and their effects may interact rather than add up. Moreover, physiological and subjective responses may diverge. A person may report feeling calm while EEG or autonomic markers suggest elevated arousal, or may experience high cognitive load during exposure to an otherwise restorative green setting. For this reason, the proposed framework should interpret urban exposure as a pattern of co-exposures and uncertainties, not as a single environmental trigger.

The recent findings from the study of urban mental health and neurourbanism confirm this statement. The use of mobile EEG has become popular for examining brain activity in urban settings, including streets, crowded places, greenery, and other environments. According to a systematic review conducted by Senkler et al. (2025), mobile EEG has increasingly been used to investigate the relationship between urban life and mental health, despite methodological heterogeneity. Likewise, according to a study by Manohar and Kurukkanari (2026), various spatial characteristics of cities may correspond to changes in stress, relaxation, involvement, and emotions.

The issues of crowding and movement within the urban environment are particularly important because they are present in the everyday lives of citizens. Mavros et al. (2022) showed that walking and crowding in both indoor and outdoor urban settings can affect psychophysiological responses, as evidenced by mobile EEG and electrodermal activity measurements. These results confirm that the brain’s response to the urban environment is not fixed. The changes depend on the type of environment, its social density, and the individual’s sensory requirements.

Different physiological markers may capture different dimensions of environmental response. EEG can provide information about attentional engagement, relaxation, fatigue, and cognitive load. At the same time, HRV may reflect autonomic regulation and recovery capacity, and EDA is especially sensitive to arousal and sympathetic activation. These signals should therefore not be treated as interchangeable indicators of stress. For example, a crowded and noisy street may increase physiological arousal and reduce perceived calm. In contrast, a visually engaging green space may alter EEG attention-related patterns without producing the same autonomic response. This is important for the proposed framework because context-aware neurofeedback should distinguish between stress-related arousal, cognitive effort, attentional engagement, and restorative recovery rather than grouping all responses under a single “stress” label (Mavros et al., 2022; Senkler et al., 2025; McDonnell et al., 2025; Vos et al., 2025).

Recent work in neuroarchitecture and environmental design neuroscience further supports the need to connect built-environment features with neural, affective, and behavioral responses. Fu et al. (2026) describe how EEG, fMRI, fNIRS, eye-tracking, and event-related potentials can provide objective measures of human responses to green buildings and built environments. This literature shows that architectural and urban design are not only physical or esthetic concerns, but also neurophysiological contexts. Yang et al. (2026) further demonstrate the relevance of linking urban environmental features with physiological health by examining mechanisms through which urban plant diversity may influence human physiological responses. The framework proposed here extends this descriptive and design-oriented literature by asking how neural, physiological, and environmental information could inform adaptive neurofeedback decisions in real time.

However, the other side of the urban exposure spectrum lies in green and restorative environments. Urban natural areas have the potential to influence stress relief, mood modulation, and recovery of mental functions. However, this would depend on the type of green space, duration of exposure, sensory stimulation, and perception of the environment. Studies by Ma et al. (2026) have revealed that various forms of city green spaces can lead to separate physiological restoration processes, in which EEGs, among other physiological measures, play an important role in identifying short-term recovery. Related experimental evidence also supports the restorative role of green exposure. Dorri Sedeh et al. (2026) found that short-term exposure to virtual urban green spaces reduced stress levels and produced physiological responses that differed from those observed in a highway environment. According to McDonnell et al. (2025), viewing natural images produced neural oscillations. This resulted in increased visual engagement but decreased cognitive processing. The findings above will play an important role in context-aware neuromodulation, as environmental exposure can have an effect of exacerbating cognitive stress or promoting neurocognitive restoration.

The field of digital exposomes links urban exposures and neuroadaptive processes. Johnson et al.’s (2023) DigitalExposome is an excellent example. In their paper, they demonstrate that sensor fusion and deep learning techniques can be applied to model the interaction between a person and the environment and to study the relationships among pollution, noise, physiological responses, and perception. However, most current research still focuses on tracking or studying the impact of exposure to the urban environment, rather than applying this knowledge to implement interactive feedback or neuromodulatory interventions. Such a disconnect is important for the field. Indeed, when the stresses of the urban environment can affect our brain and body states, AI-powered neuromodulation cannot depend solely on neural biomarkers but should also consider the environmental context in which these biomarkers arise. Is it in a busy street, a heat-stressed neighborhood, a polluted road, or a restorative green zone?

Urbanization is viewed as an active layer in neuromodulation within the model. Urban exposure is not just information that provides context for brain states; it also becomes part of the context for interpreting brain readings. Including environmental exposure measures with EEG and physiological sensors will help future models to distinguish internal variations, exposure to stressors, and recovery environments.

## 4 Spatial intelligence and environmental exposure modeling

For context-aware neuromodulation to function beyond the laboratory, urban exposure must be translated into measurable and interpretable variables. Spatial intelligence is the solution. It refers to the use of geographic information systems, environmental mapping, remote sensing, spatial classification, and location-based data to describe how individuals are subjected to different environmental conditions throughout urban space. In the present model, spatial intelligence is used not only to map the city but also to transform it into a clearly structured input layer for AI-based interpretation of brain and body states.

GIS can capture a lot of what shapes urban neurophysiological responses—land use, density, road layout, green space, surface materials, proximity to water, pollution hotspots, and noise levels. Paired with sensing data, these variables help explain what is actually behind an EEG or physiological signal, whether that is traffic noise, heat, crowding, or the loss (or restoration) of green space. Zhang et al. (2024), for instance, used machine learning on street-level imagery and found a nonlinear relationship between green space exposure and perceived stress.

LCZ classification is particularly useful here. It describes both built and natural areas in terms of surface cover, building form, density, height, and thermal properties. This provides researchers with a standard way to model urban morphology and climate exposure. Reviews point to LCZ as a solid tool for studying urban climate, outdoor thermal comfort, and city planning (Han et al., 2024). In the proposed approach, LCZs can serve as variables for spatial context. For example, compact high-rise, compact low-rise, open low-rise, industrial, green, or water can serve as various exposure contexts. These can be used in combination with environmental variables including temperature, humidity, noise, particulates, traffic, and access to green space. It will be viewed as an exposure context that may affect stress, attention, and cognitive load, instead of thinking of location as simply a geographic coordinate.

LCZs should not be treated as the only spatial unit of analysis. In the proposed framework, LCZs provide a macro- or meso-scale background layer describing urban morphology. Micro-scale indicators capture the immediate sensory environment. These may include shade from street trees, local surface temperature, traffic proximity, sound level, visible greenery, pedestrian density, and momentary exposure to sunlight or wind. EEG and physiological data should therefore be aligned with both slow-changing spatial descriptors and fast-changing micro-environmental measurements. This multi-scale structure helps reconcile relatively static GIS information with high-frequency human sensing data.

Literature shows that this type of integration is already possible. Johnson et al. (2023) demonstrated that urban environmental data, physiological responses, perceived experience, timestamps, and geotagged information can be combined through sensor fusion and deep learning. Similarly, the Leef model highlights the value of linking environmental and physiological data to estimate cognitive load in real-world settings (Morina, 2026). However, the framework proposed here takes this idea further by connecting spatial exposure modeling with adaptive neurofeedback and neuromodulation. Existing digital exposome and environmental neuroscience approaches mainly focus on measuring exposure, wellbeing, or cognitive load. In contrast, a context-aware neuromodulation system would use spatial exposure as part of the feedback decision process. For example, high arousal in a busy traffic corridor may require a different interpretation than the same arousal level in a quiet green space.

Several environmental variables could also be part of this layer. Static variables would include the LCZ classification, building density, land cover, green space coverage, proximity to roads, and proximity to parks and water bodies. Dynamic variables would include air temperature, humidity, noise, air quality, crowding, mobility speed, time of day, and routes. Other factors such as perceived stress, comfort, safety, and environmental preference could also be considered. This spatial layer is needed because context-sensitive neuromodulation requires not only real-time classification of brain state via EEG but also awareness of the circumstances that promote such states. Spatial intelligence through GIS can serve as the system’s environmental memory. EEG and physiological sensing provide real-time data about the person. Through their mixing, the system can progress from simply modeling brain states to modeling brain–body-environment states.

By leveraging spatial intelligence, the model integrates urban planning, environmental well-being, and neurotechnology. This approach will also provide a viable path for future non-clinical uses of technology. Applications can include adaptive neurofeedback for urban movement, personal stress-recovery suggestions, route-based exposure, and even mental well-being in the smart-city setting. The following section explains the proposed AI framework.

## 5 Proposed AI framework for context-aware neuromodulation

The suggested model integrates brain state tracking, physiologic sensing, environmental exposure, and spatial intelligence into a single adaptive model. The main aim of this framework is not to replace traditional neuromodulation and neurofeedback technologies, but rather to augment them by incorporating the urban environment in which brain and physiological states arise. In this framework, the person is not considered an independent biological system. Instead, brain activity, physiologic regulation, and environmental exposure are seen as the components of a brain–body-environment system.

This architecture includes four different interacting layers: the environmental exposure layer, the human sensing layer, the AI fusion and interpretation layer, and the adaptive neurofeedback/neuromodulation layer. The environmental exposure layer captures the environmental conditions from the spatial and physical context in which the user exists. This can involve various factors such as Local Climate Zone classification, building density, road proximity, traffic levels, accessibility to green spaces, temperatures, humidity, air pollution, noise, crowding, and time of day. Such factors can be captured using GIS data, remote sensing, environmental sensors, mobility traces, or urban exposure models. This layer provides information on the user’s location and the environmental load that may be present there (Cenameri and Albert, 2021; Han et al., 2024; Johnson et al., 2023).

The human sensing layer detects the user’s current neurophysiological status. Information about oscillations related to attention, relaxation, load, tiredness, or arousal could be detected using EEG. At the same time, physiologic measures, such as heart rate variability, electrodermal activity, body movements, and sleep data, can supply additional information about autonomic nervous system functioning and recovery. With recent developments in portable EEG devices, such as in-ear EEG and embedded machine learning in wearable EEG, it seems increasingly realistic to monitor those parameters even outside experimental conditions (Channa et al., 2026). The human sensing layer is crucial because environmental stimuli alone cannot determine a response. The same environment can induce different responses due to a person’s internal state.

The AI fusion and interpretation layer incorporates data from both environmental and human sensing layers. Machine learning, deep learning, multimodal fusion, or probabilistic models can be used in this layer to detect common patterns within EEG data, physiological measures, and environmental exposure parameters. The purpose of this layer is not only to categorize the state of the brain but to interpret it in the context of the environment. The high level of arousal, coupled with high noise, heat exposure, and traffic density, would be interpreted differently from the high level of arousal during active walking in a green area.

### 5.1 Methodological blueprint for multimodal AI fusion

The practical application of the fusion layer in AI would involve a multistep analytical process as opposed to a monolithic black-box model. The first step is to segment the EEG, HRV, EDA, movements, and self-report into shorter time windows of about 10–60 s, depending on the state of interest and the quality of the signal. Second, each window should be annotated with the spatial and environmental information about the location, which could include LCZ classification, green space, traffic, noise, heat, air pollution, and overcrowding. Recent studies have shown the efficiency of AI-enabled closed-loop models in detection, prediction, and decision-making, as well as the accuracy of neuromodulatory technologies (Yang et al., 2024).

Several methods might be utilized, depending on the nature of the research question and size of the dataset. Generalized Additive Models can be used to test non-linear exposure-response relationships. Also, to find thresholds of restoration or stress. Recent green space research studies indicate that the effects of exposure can follow a threshold principle rather than a linear one (Dai et al., 2026). The Structural Equation Model is useful in studying the cause-and-effect relationship between environmental exposure, perception of environmental risk, arousal, and EEG indices (Yang et al., 2026). Other models like probabilistic or Bayesian models could capture uncertainty in situations where it is hard to distinguish between internal and external stressors. Deep-learning models may be useful in larger datasets, but should be introduced only after adequate validation and interpretability procedures. The output of the system should therefore be a probabilistic context-aware state estimate rather than a deterministic label.

The adaptive neurofeedback or neuromodulation component takes the interpreted state into account to generate an adequate response. For example, in non-clinical scenarios, such a response can consist of EEG neurofeedback, breathing instructions, relaxation tips, an adaptive soundscape, visual cues, attention training, or even route-specific recovery suggestions. The same architecture in future controlled use cases might guide more sophisticated neuromodulation procedures; however, the current approach makes no assumptions about clinical intervention or autonomous stimulation. The critical point is that any feedback needs to be adapted to both the user’s brain state and the environment in which it occurs.

This model has been inspired by the principle of closed-loop neuromodulation, where real-time monitoring is used for real-time intervention. Recent studies have shown the effectiveness of AI-based closed-loop models for the purposes of detection, prediction, and decision making and the accuracy of neuromodulatory technologies (Yang et al., 2024). In addition, neurofeedback studies demonstrate that real-time feedback may assist in learning to modulate neural activity, but only when appropriate training and feedback protocols are used (Galang et al., 2025). The presented framework incorporates an additional environmental layer into the feedback loop. In this sense, the environmental layer functions as a contextual constraint on interpretation rather than as a deterministic explanation of the user’s state.

The operational process can be summarized as follows. Firstly, the system determines the user’s context using GIS technology, environmental sensors, or location-related environmental exposures. Secondly, the system captures EEG and physiological data to determine the user’s internal state. Thirdly, the artificial intelligence component integrates the inputs to assess whether the user experiences cognitive load, stress-related arousal, recovery, or attentional instability. Fourthly, the feedback mechanism corresponding to both internal and external conditions is selected.

**Figure 1:**
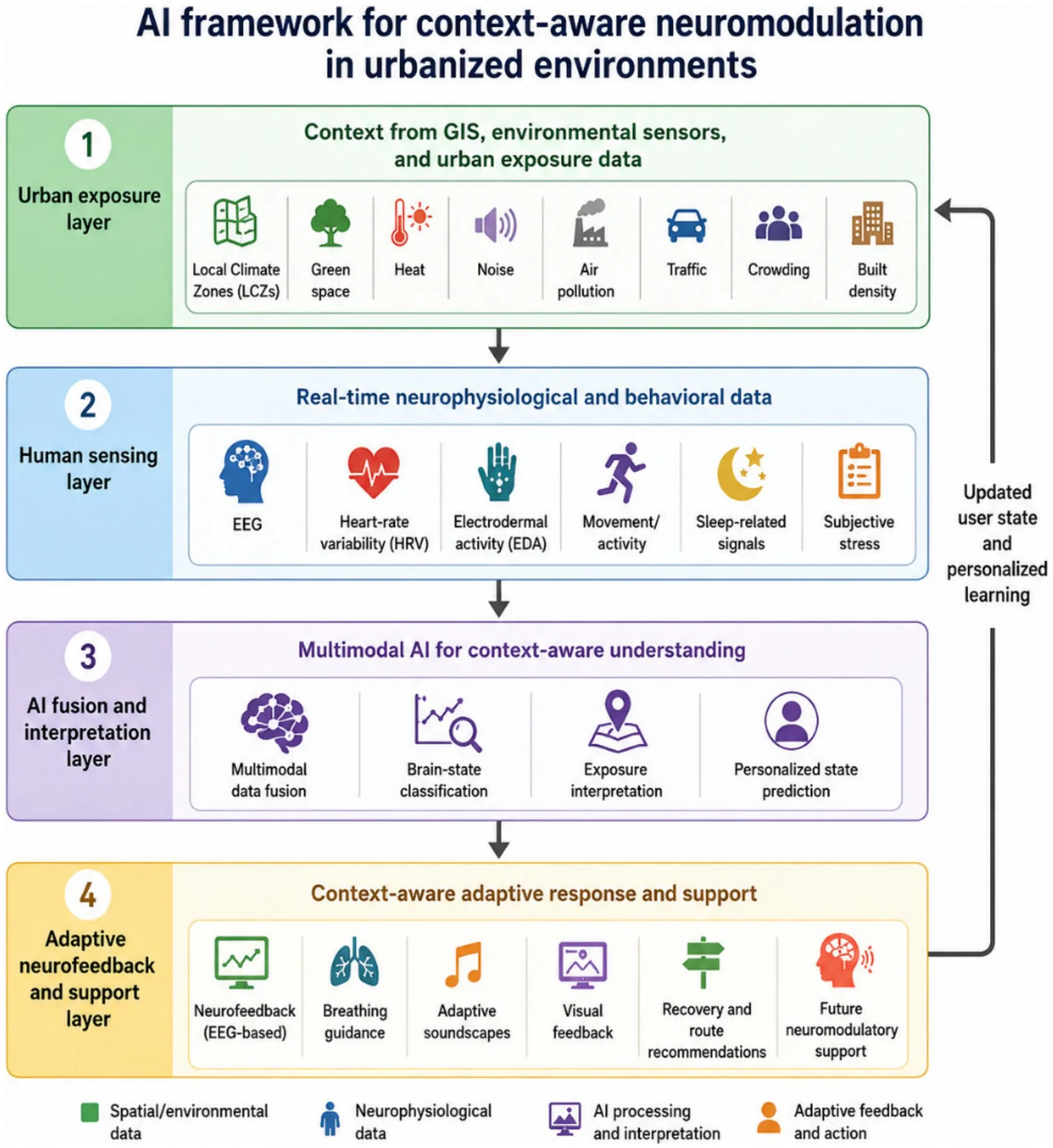
AI framework for context-aware neuromodulation in urbanized environments.

The novelty of the proposed framework lies in treating urban environmental exposure as an informative input rather than as background noise in adaptive neurotechnology. Urban conditions such as heat, crowding, noise, traffic density, and vegetation are considered contextual variables that may help interpret EEG and physiological responses more accurately. It should be understood as conceptual and future-oriented. It outlines a research direction that connects AI-driven neuromodulation, mobile EEG, physiological monitoring, digital exposome modeling, and GIS-based spatial intelligence (Figure 1).

## 6 Use-case scenario

This section provides a case study that is not a validated application. It demonstrates how context-aware neuromodulation can function.

Let us think of a person traveling through a crowded city. A crowded public transportation station, a shaded pedestrian street, a tiny urban park, and a narrow traffic corridor with high noise and heat exposure are among the exposure contexts along the route. In a traditional EEG-based neurofeedback system, the user’s brain activity may be largely interpreted in terms of internal neural properties, such as variations in alpha, beta, or theta activity. At the beginning, the spatial layer indicates that the individual is in a high-density built environment characterized by a lack of vegetation cover, heavy traffic, and potential heat. The human sensor layer monitors the individual’s brain waves (EEG) and other physiological data, such as heart rate variability, electrodermal activity, and movement. If the AI fusion layer detects signs of arousal or relaxation inhibition at this stage, it does not treat them as independent states of mind. It checks whether there is any connection to the surrounding urban environment, such as traffic, heat, crowding, or visual complexity.

As the user enters the congested transport station, the device can detect physiological arousal. Research involving portable EEG studies in urban settings indicates that congestion, movement, and the urban environment affect arousal and attention levels (Mavros et al., 2022; Senkler et al., 2025). In this case, the model may classify the state as probable exposure-related cognitive load or stress-related arousal. However, feedback intensity should depend on situational safety. In high-density traffic areas, the system should avoid visually demanding or cognitively distracting feedback. Safer options may include passive monitoring, minimal haptic cues, auditory cues that do not mask environmental sounds, or delayed feedback after the user reaches a safer location. Adaptive feedback should therefore be context-aware not only in relation to stress or recovery, but also in relation to safety.

**Figure 2:**
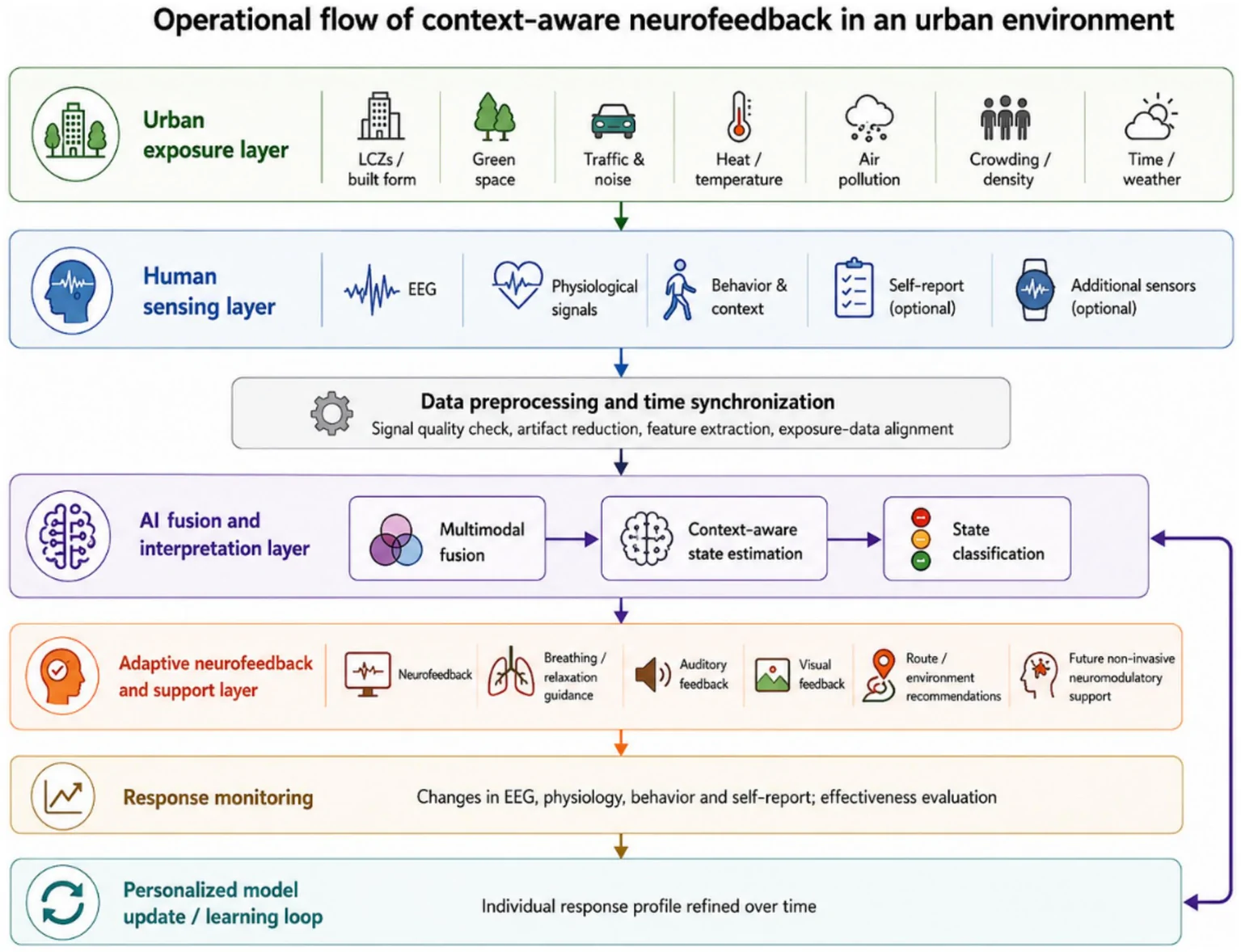
Operational flow of context-aware neurofeedback in an urban environment.

When the user later steps into an area with shade and greenery or an urban park, the environmental level is altered. At this point, the system identifies increased exposure to green space, reduced traffic density, and possible noise reduction. According to scientific research on green spaces in cities, exposure to green space can be conducive to stress recovery and restore an individual’s physiology. However, it will depend on the quality of such an environment (Ma et al., 2026; Dorri Sedeh et al., 2026). As signals from EEG and physiological indicators shift toward relaxation and recovery, the AI model may adjust the user’s state and reduce feedback. So, the system not only detects stress but also recovery conditions. Figure 2 illustrates the process described above.

The system should not assume that arousal is always environmentally induced. If a user shows elevated arousal while walking through a green space, the model should consider competing explanations such as internal worry, a stressful phone call, physical exertion, time pressure, or social interaction. In such cases, the output should be treated as uncertain. Rather than assigning a strong label, the system could provide a low-burden suggestion, delay feedback, or request optional user confirmation.

In the long run, patterns formed from observations might result in individualizing the system’s feedback strategy. For instance, while one user tends to experience a higher level of physiological arousal in transport hubs, another may be more vulnerable to exposure to heat and traffic noise. A contextualized approach would enable the system to understand when it needs to provide breathing instructions, awareness feedback, or even suggest routes to recover. This personalization process aligns with the overall trend toward adaptation and neuromodulation, aided by AI (Yang et al., 2024; Galang et al., 2025).

Such an example also demonstrates the significance of spatial intelligence. In the absence of spatial and environmental information, it may detect arousal but still be unsure of its potential context. Using spatial exposure modeling, it will be able to discriminate between arousal in the context of a traffic-filled street, engagement that happens when walking actively, and recovery in a natural environment. It does not imply that the environment influences the state of the brain directly. Instead, the role of the environment is in interpretation.

From a practical perspective, this approach would involve the use of a wearable EEG headset, physiology sensors, an app, and a GIS-based exposure model. The importance of wearable EEG and embedded intelligence is growing for ambulatory brain-state estimation, while the digital exposome framework has already demonstrated how geotagged data on both environment and physiology could be used in an urban area (Channa et al., 2026; Johnson et al., 2023). Nevertheless, it should not be forgotten that privacy and ethical aspects of data collection and utilization need to be considered carefully to follow regulations and protect the rights of users.

While still conceptual, the use case shows the true value of the system. To move toward practical realization, it is necessary to develop validation procedures that demonstrate the system’s effectiveness in real urban settings. Context-aware neuromodulation does not react just to the brain signal. It reacts to the brain signal as presented in the particular urban exposure situation. The shift from isolated brain-signal analysis to situational analysis is crucial for making neuromodulation and neurofeedback systems relevant in urban conditions.

## 7 Discussion

### 7.1 Theoretical contribution and interdisciplinary position

The framework benefits adaptive neurotechnology in the sense that it changes the approach toward interpreting brain signals alone to inferring brain–body-environment relations. This is crucial since neurophysiological signals are not generated in isolation. Within the urban environment, these signals are affected by exposures such as heat, traffic noise, overcrowding, air pollution, visual stimuli, and availability of restorative environments. So, the primary contribution of the proposed framework does not lie in the incorporation of the environmental measures but in using these data to enhance understanding of neurophysiological conditions.

This research builds upon existing literature on closed-loop neuromodulation and neurofeedback, where brain state tracking, biomarkers, and machine learning algorithms have been increasingly adopted to enhance adaptive intervention logic (Yang et al., 2024; Kachhadia et al., 2025). In addition, it is informed by existing literature on digital exposome and environmental neuroscience research in exploring the possibility of utilizing exposure information in adaptive feedback. The application of machine learning in decision-making in AI-supported neuromodulation and chronic pain is evident in clinical research. However, such clinical applications cannot necessarily be generalized to urban neurofeedback applications (Siddiqi et al., 2026).

The model is also relevant to the study of urban public health and smart cities. The greenspace literature and the urban green exercise literature indicate that environmental design may promote mental well-being and mental recovery by improving access to restorative environments (Ma et al., 2026; Hu et al., 2025). Nonetheless, context-aware neurofeedback must not be used instead of urban planning and public health interventions but rather complement them.

### 7.2 Ethical, technical, and societal challenges

Context-aware neuromodulation raises several ethical, technical, and societal challenges that must be addressed before real-world implementation. The central challenge will be the one of data privacy and management because gathering EEG, physiological, mobility, environmental, and stress data could give away some private information regarding the person’s psychological condition and vulnerabilities. The combination of such data makes it even more vulnerable than single data types. In the future, there should be mechanisms of data minimization, local computing, anonymization, data sharing limitations, and consent.

Reliability of technical aspects is another important issue. The actual EEG is influenced by the following factors such as motion artifacts, electrode attachment, muscular activity, electromagnetic interference, and equipment-related problems. These problems are particularly important in the city environment since people will be walking, traveling, and even experiencing heat or noise. It should also be mentioned that mobile EEG research is also heterogeneous concerning protocols, definitions of exposure, data preprocessing and sample size (Senkler et al., 2025). Low-cost EEG studies are also restricted by certain factors (Vos et al., 2025).

Also, multimodal synchronization can be considered a challenge. EEG, physiological signals, GPS, environmental indicators, and subjective reports differ in temporal resolution, spatial precision, latency, and uncertainty. A further difficulty is that some exposure variables are categorical or slowly changing, such as LCZ class or land-use type. In contrast, EEG, HRV, and EDA are continuous time-series signals. Without careful alignment, AI models may learn misleading links between environmental context and brain–body responses. For this reason, preprocessing, signal-quality control, time synchronization, and uncertainty estimation should be treated as core parts of the framework.

The fourth issue is interpretability. An AI-based neuromodulation platform might use machine learning or deep learning to infer brain states and select a feedback strategy. However, the inability of such a platform to explain how a given brain state was inferred or why it chose a particular feedback strategy could negatively affect user confidence and scientific credibility. In the case where exposure to the environment is considered, such a platform may wrongly attribute arousal to exposure to noise, warmth, overcrowding, or green space deficiency.

The fifth issue arises due to the danger of overgeneralization. The same environmental exposure triggers different reactions in different people. For example, being on a crowded street can be stressful for some, exciting for others, and neither here nor there for some other individual. Likewise, while green spaces are often associated with recovery, the effect may vary depending on factors such as safety, accessibility, esthetic perception, prior experience, and preferences. Hence, the framework should be individualized and probabilistic rather than universal and deterministic.

The sixth challenge relates to the distinction between non-clinical support and clinical treatment. The current framework is intended as an idea for a non-clinical adaptive neurofeedback system and for future neuromodulation assistance. It cannot be introduced as an aid to diagnosis or therapy until it has been empirically proven. In the field of clinical neuromodulation, including artificial intelligence for pain control, machine learning can increase the quality of decision-making and forecasting. Still, such practices must undergo rigorous validation, safety checks, and clinical approval (Siddiqi et al., 2026). This caution should also be exercised in the field of urban neurofeedback.

**Figure 3:**
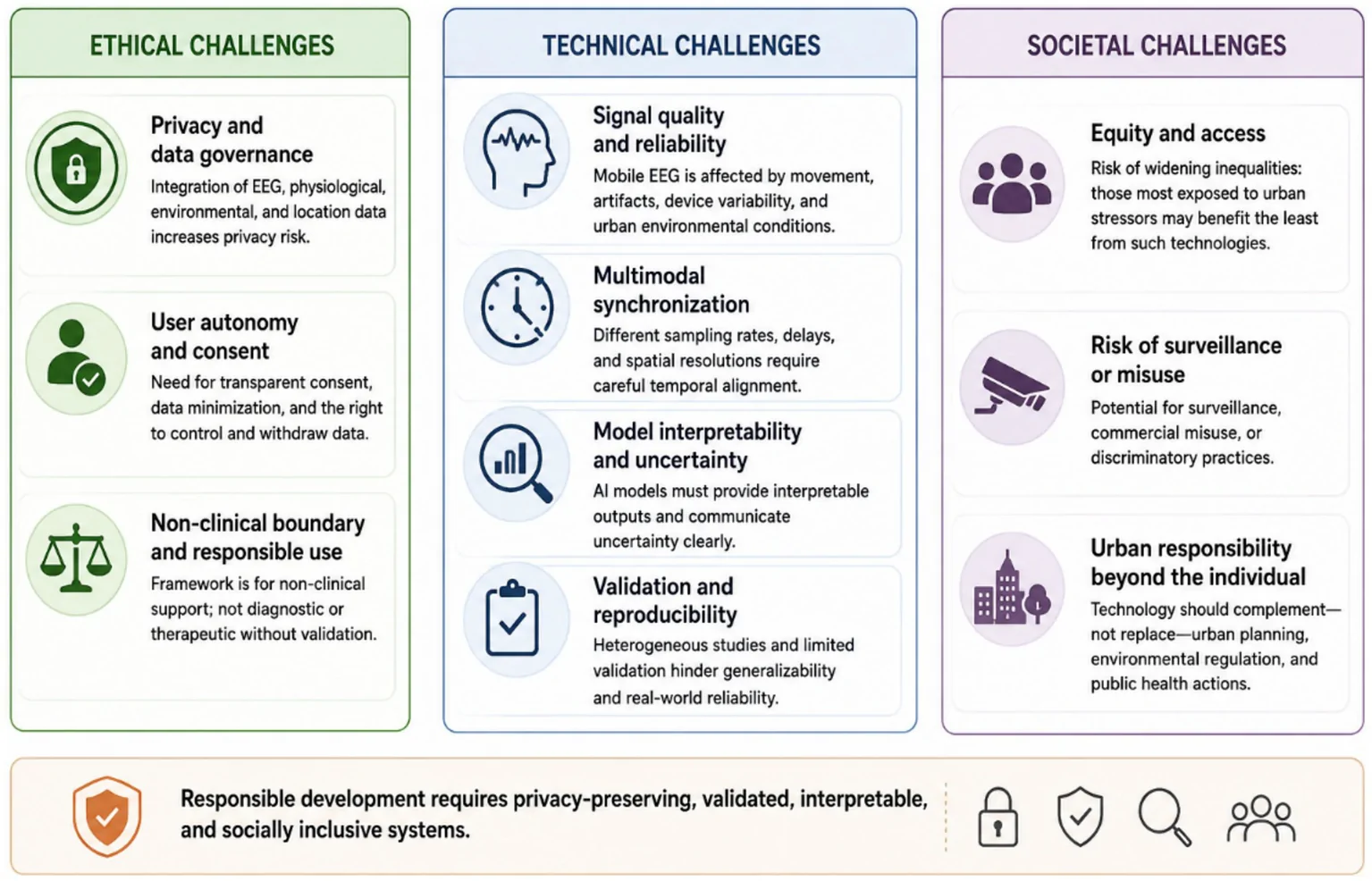
Ethical, technical, and societal challenges of context-aware neuromodulation.

Continuous monitoring and feedback may help some users understand their stress and recovery patterns, but it may also increase self-monitoring anxiety or shift responsibility toward individuals. A system that prompts users to regulate breathing in a noisy, polluted, or heat-exposed street should not imply that individuals are responsible for adapting to conditions created by poor urban design. Context-aware neurofeedback should support self-regulation, but it should not replace public health, environmental regulation, or urban planning. Figure 3 summarizes the main ethical, technical, and societal challenges discussed in this section.

There are societal and equity issues. Neurotechnology and AI systems may be more accessible to the wealthy, leaving vulnerable populations with little benefit. Implementing these on smart city platforms could raise concerns about surveillance and data misuse. The framework should be privacy-preserving, sound, interpretable, nondeterministic, and socially responsible. Its future depends on AI performance and its capacity to protect and empower users.

### 7.3 Future research directions

The framework is a starting point for future research. While integrating EEG, physiological sensing, environmental exposure, and spatial intelligence is promising, several steps are needed before reliable context-aware neuromodulation can be implemented. First, standardized protocols for urban neurophysiological experiments must be developed. Currently, mobile EEG studies vary widely in sample size, recording conditions, exposure characterization, signal processing, and outcomes (Senkler et al., 2025). Future studies should define urban exposures more precisely, such as traffic, noise, heat, green space, crowding, and density. This will produce more consistent results and promote AI training. Empirical testing could compare the accuracy of exposure-informed models with that of exposure-naive models using the same signals.

Secondly, the generation of multimodal datasets incorporating brain, body, behavior, and environment could be a focus of future research. This type of dataset would require incorporating EEG, heart rate variability, electrodermal activity, movement data, self-reports, GPS paths, environmental sensor data, and GIS measures of exposure. Digital exposome studies have already shown the usefulness of incorporating such information in urban environments (Johnson et al., 2023). The next step for future research would then be to incorporate EEG and other neurofeedback outcomes to predict brain–body states, as well as exposure and well-being outcomes.

The third line of work involves enhancing the spatial-exposure layer. Local climate zones, green space, proximity to roads, heat exposure, and land use attributes can give a systematic description of the urban environment (Cenameri and Albert, 2021; Han et al., 2024; Gál et al., 2026). But future studies should decide what spatial features can be helpful in the interpretation of neural and physiological responses. Not all variables from GIS are suitable for neuromodulation. Studies need to identify which predictors are important for stress-induced arousal, cognitive load, attentional recovery, and relaxation.

Fourthly, there is a need to develop personalized models of AI. General models may be used in identifying general trends. However, context-aware neurofeedback will definitely require personalization. They must consider individual differences in stress levels, environment, mobility patterns, physiological baselines, and the ability to control brain states via feedback. In the future, AI will need to incorporate the patterns identified at the population level and those learned at the individual level.

The fifth line concerns the verification of feedback approaches. It is insufficient to label a state as one related to arousal or recovery. For example, there also needs to be an evaluation of what kind of response will be helpful. Researchers need to test several kinds of feedback in future experiments, such as EEG neurofeedback, breathing, auditory feedback, visual feedback, routes, or combinations thereof. As shown by neurofeedback research, training design, target signals, and other aspects of feedback play an important role in the development of neural modulation (Galang et al., 2025). For context-aware systems, all these aspects need to be verified in different environments. Future research should also develop threshold models that define when environmental exposure shifts from stressor to restorative condition. These thresholds should be tested using EEG, HRV, EDA, subjective restoration scores, and micro-environmental indicators such as green-view index, shade, noise, temperature, and crowding. Recent greenspace studies suggest that physiological and psychological benefits may emerge within short exposure windows and may follow non-linear or threshold-based patterns rather than simple linear improvement (Yao et al., 2023; Yang et al., 2025; Dai et al., 2026). Establishing such thresholds is necessary before an AI system can responsibly select personalized feedback based on restorative potential.

The sixth area relates to wearable and edge-computing solutions. In-ear EEG devices and other wearable solutions have become increasingly applicable to the study of brain states on the go, but their use in regular urban settings must be validated carefully (Channa et al., 2026). In future systems, the emphasis must be put on maintaining signal quality, minimizing power consumption, conducting data analysis locally, and developing privacy-preserving models. Edge computing can be especially important because it can help avoid uploading EEG and physiological data to servers.

Another direction is that of longitudinal research. Most research today looks at the impact of acute reactions to urban environments on people (Senkler et al., 2025), but stress and adaptation occur over time. Longitudinal research could look at whether there are consistent neurophysiological markers because of repeated exposure to certain urban environments, how the feedback leads to a change in self-regulation over weeks or months, and how adaptation leads to more effective recovery.

The eighth aspect of concern is that of user experience and participatory design. The context-aware neuromodulation technology should not only be developed in terms of its functionality. It must also be considered in accordance with the needs of the user. In this regard, the user must be taken into consideration when setting acceptable feedback frequencies, privacy preferences, data sharing, and the type of assistance the user may require. This will be especially crucial given the psychological pressure associated with constant monitoring.

A final direction concerns the link between context-aware neurofeedback, urban planning, and public health. Such systems should not be developed only as tools for individual self-regulation. If aggregated and processed in privacy-preserving ways, their outputs could help identify urban areas that are consistently associated with higher stress burden or lower recovery potential. However, any use beyond the individual level would require strict ethical governance, transparency, and safeguards against surveillance or profiling. The broader aim should be to support healthier urban environments while protecting individual rights.

In summary, future research needs to advance step by step. It starts with conceptual models, moves to pilot tests, then to field validation, and eventually to appropriate applications. More important than simply asking whether AI can identify the various brain states correctly is asking whether it is also possible for AI to understand the relationship of such states with the environment in which humans operate.

## 8 Conclusion

The paper suggested a conceptual model of context-aware neuromodulation in urban environments. The main thesis of this paper is that in the future, AI-powered neurofeedback and neuromodulatory systems should not consider brain activity as a standalone signal but rather should consider the brain state in connection with physiological, behavioral, environmental, and spatial contexts.

The framework consists of four key components: urban exposure modeling, human sensing, AI-driven multimodal analysis, and adaptive neurofeedback/neuromodulation support. The framework shifts from brain-only adaptation to brain–body-environment adaptation through the integration of EEG, physiological measurements, spatial intelligence through GIS, environmental measurements, and personalized learning. In particular, the concept of brain–body-environment adaptation becomes increasingly significant within urban settings due to the constant impact of traffic, noise, air pollution, heat, crowding, density, greenery, and mobility on stress, cognitive load, attention, and recovery.

The innovation of this model is that it treats urban exposure as an active contextual layer rather than just background data. Spatial intelligence in the form of Local Climate Zones and GIS exposure indices can help transform urban environments into quantifiable elements for artificial intelligence to interpret. Thus, a neural or physiological pattern can be analyzed not only based on the characteristics of its signal but also on the environment where it happens. With proper development, these technologies can be used for self-regulation and stress awareness, as well as future applications, while also informing larger conversations about urban health and exposure.

However, the model itself is conceptual and futuristic. It does not make claims about being clinically validated, diagnosable, or applicable in therapy. There are still many challenges to overcome regarding data standardization, reliability of EEG readings in wearable devices, multimodal synchronization, model interpretability, privacy preservation, and ethical management. These limitations are critical.

## Data Availability

The original contributions presented in the study are included in the article/supplementary material, further inquiries can be directed to the corresponding author.
